# Childhood cancers in a referral hospital in northern Nigeria

**DOI:** 10.4103/0971-5851.64253

**Published:** 2009

**Authors:** A. Mohammed, H. O. Aliyu

**Affiliations:** *Department of Pathology, Ahmadu Bello University Teaching Hospital, Shika-Zaria, Nigeria*

**Keywords:** *Childhood cancers*, *lymphoma*, *Nigeria*

## Abstract

**Background:**

We undertook this study to determine the relative frequencies of childhood malignancies and their age – sex distribution in this environment.

**Materials and Methods:**

Hospital-based data of histological and cytologically confirmed cases of malignancies in children, aged ≤ 15 years, was collated over a period of 11 years, from the Cancer Registry.

**Results:**

A total of 329 children aged ≤ 15 years, with confirmed malignant disease, was recorded. This constituted 8.44% of all malignancies diagnosed in the same period with a Male : Female ratio of 1.5:1. Burkitt’s lymphoma accounted for 27.01% of the cases followed by retinoblastoma (17.02%), non-Hodgkin’s non-Burkitt’s Lymphoma (9.42%), and Rhabdomyosarcoma (9.42%). Others were Nephroblastoma (8.81%), Hodgkin’s lymphoma (6.69%), Neuroblastoma (3.34%), Colorectal carcinoma (2.43%), Osteosarcoma (2.13%), and Unspecified lymphomas (1.82%). Burkitt’s lymphoma was most prevalent in the 5–9 and 10–15 year age groups, retinoblastoma in the 0–4 year age group, and Non–Hodgkin’s lymphoma, Hodgkin’s lymphoma, and unspecified carcinomas were more prevalent in the 10–15 year age group.

**Conclusion:**

Lymphomas were the most prevalent malignancies of childhood seen in this region and the majority were of the Burkitt type, in contrast to the predominant leukemic and central nervous system trend seen in developed nations.

## INTRODUCTION

The emerging threat of cancers in developing countries, especially in the pediatric age group, has received little attention. This can be explained by the preoccupation with infectious diseases, such as malaria, which contributes to about 25% of the deaths in children under the age of one.[1] However there are some growing concerns on the incidence and management of childhood cancers in tropical African countries.[2,3] Studies of cancer in children have contributed greatly to the understanding of the genetic processes involved in carcinogenesis.[4,5] As there are peculiarities and variations in the occurrence of specific cancers with respect to age, sex, race and so on, it was considered appropriate to undertake this retrospective study, with a view to determining the most common malignancies in children aged ≤ 15 years, including their age and sex distribution in this environment. This will form a basis for comparison with studies from different parts of the country and other parts of the world. The report may also help relevant institutions focus on the planning of intervention programs.

## MATERIALS AND METHODS

Ahmadu Bello University Teaching Hospital (ABUTH) is a teaching and referral facility located in the north central region of Nigeria. Most cases of childhood and adult cancers in the northern region are referred here for confirmation of diagnosis and / or treatment.

Using predesigned data sheets, details on all children aged ≤ 15 years, diagnosed with malignant conditions, was extracted from the cancer registry of the Hospital for the period January 1994 to December 2004. The information retrieved was verified against the admission registers and laboratory registers kept by the hospital’s Records Department. All cases with inconsistencies were excluded from the study (there were three cases of non-statement of the child’s sex in the records). The sex and age distribution of the different malignancies were described using percentages and relevant data displayed in Tables. The International Classification of Childhood Cancers (ICCC) was used.[6]

## RESULTS

A total of 3899 malignancies were diagnosed over the 11-year period (1994 – 2004) and of these, 329 cases were in children ≤ 15 years, constituting 8.44% of all the cases. Using the ICCC, the malignancies seen over this period are outlined in Table 1. This shows the age – sex distribution of all cancers diagnosed during the period. Overall, Burkitt’s lymphoma, retinoblastoma, non-Hodgkin’s lymphoma, and rhabdomyosarcoma accounted for 59.88% of all tumors recorded over the study period. Burkitt’s lymphoma accounted for 27.01% of the cases, followed by retinoblastoma (17.02%), non-Hodgkin’s non-Burkitt’s Lymphoma (9.42%), and Rhabdomyosarcoma (9.42%). Others were Nephroblastoma (8.81%), Hodgkin’s lymphoma (6.69%), Neuroblastoma (3.34%), Colorectal carcinoma (2.43%), Osteosarcoma (2.13%), and unspecified lymphomas (1.82%). Table 2 gives the sex distribution of the ten most common malignancies in each age group.

**Table 1:** Age–Sex distribution of childhood cancers in the Zaria cancer registry (1994–2004)

ICCC		N	Male	Female	M/F ratio	Relative frequency %
I	Leukemia				1:2	
	ALL	2	0	2	–	0.60
	Unspecified	1	1	0	–	0.30
II	Lymphoma					
	Hodgkins	22	19	3	6.3:1	6.69
	Non-Hodgkins non-Burkitt’s	31	24	7	3.4:1	9.42
	Burkitt’s	79	55	24	2.3:1	24.01
	Others specified	1	0	1	–	0.30
	Others non-specified	6	4	2	2:1	1.82
III	CNS, Intracranial, Intraspinal neoplasms	0	0	0	–	
IV	Neuroblastoma	11	4	7	1:1.8	3.34
V	Retinoblastoma	56	27	29	1:1.1	17.02
VI	Nephroblastoma	29	17	12	1.4:1	8.81
VII	Hepatoblastoma	1	0	1	–	0.30
VIII	Bone Tumor					
	Osteosarcoma	7	2	5	1:2.5	2.13
	hondrosarcoma	1	1	0	–	0.30
	Ewing’s sarcoma	2	2	0	–	0.61
IX	Soft tissue					
	Rhabdomyosarcoma	31	17	14	1.2:1	9.42
	Neurofibrosarcoma	1	1	0	–	0.30
	Kaposi Sarcoma	3	2	1	2:1	0.91
	Others	2	1	1	1:1	0.61
X	Germ cell/gonadal					
	Sacrococcygeal tumor	3	0	3	–	0.91
	Testicular tumor	1	1	0	–	0.30
	Ovarian tumor	2	0	2	–	0.61
XI	Carcinomas					
	Nasopharyngeal	2	2	0	–	0.61
	Skin	4	2	2	1:1	1.22
	Colorectal carcinoma	8	5	3	1.6:1	2.43
	Conjuctival tumors	5	3	2	1.5:1	1.52
	Thyroid tumors	1	0	1	–	0.30
	Salivary gland tumors	1	0	1	–	0.30
	Genitourinary tumors	2	2	0	–	0.61
	Endometrial tumors	1	0	1	–	0.30
	Oral tumors	1	0	1	–	0.30
	Breast	1	0	1	–	0.30
	Unspecified	2	1	1	1:1	0.61
XII	Other malignant tumors					
	Specified	3	1	2	1:2	0.91
	Unspecified	6	1	5	1:5	1.82
Total		329	196	133		100.00
			59.57%	40.43%		

**Table 2:** Sex distribution of the ten most common malignancies in children from the Zaria cancer registry (1994–2004)

Tumours	Age group						Total	Percentage
	0 – 4		5 – 9		10 – 15			
	M	F	M	F	M	F		
Burkitt’s lymphoma	4	3	37	15	14	6	79	24.01
Retinoblastoma	17	23	9	5	1	1	56	17.02
Non-Hodgkins non-Burkitt’s lymphoma	2	1	7	4	15	2	31	9.42
Rhabdomyosarcoma	6	8	5	2	6	4	31	9.42
Nephroblastoma	11	9	4	2	2	1	29	8.81
Hodgkins lymphoma	1	0	5	0	13	3	22	6.69
Neuroblastoma	2	3	0	3	2	1	11	3.34
Colorectal carcinoma	1	0	0	0	4	3	8	2.43
Osteosarcoma	0	0	1	0	1	5	7	2.13
Unspecified lymphomas	0	0	4	0	0	2	6	1.82
Total/Percent	44	47	72	29	58	24	280	85.09

## DISCUSSION

This study presents 8.44% of all malignancies as the contribution by childhood cancers. An earlier study[7] from this center has reported 5.6%, although the present study covers a longer period and has a higher population of children. Studies from Cuba[8] report a figure of 13.06%, while those from Ibadan[8] and Pakistan[9] report 4.3 and 12.5%, respectively. This relatively wide variation may be attributable to peculiarities in the types of cancer that predominate. The present study shows a predominance of Burkitt’s lymphoma, and in decending order retinoblastoma, non-Hodgkin’s lymphoma, rhabdomyosarcoma, nephroblastoma, Hodgkin’s lymphoma, and carcinomas. This picture is similar to the earlier study[7] carried out in this center, but varies remarkably from the Cuban study,[8] where leukemia and central nervous tissue cancers predominate. Although leukemia in our study shows a relatively low frequency of 0.91%, many cases may have gone unreported.

Burkitt’s lymphoma was most prevalent in the 5 – 9 and 10 – 15 years age groups, while retinoblastoma was prevalent between the 0 – 4 years age group. This is similar to other reports.[2,3,7] Burkitt’s lymphoma has been a recurrent and highly prevalent cancer in our environment.

This study shows male preponderance. This is similar to other studies,[7,9] and the Cuban study[8] showed an equal ratio. Considering individual cancers, Burkitt’s lymphoma shows a predominance of the male gender. Retinoblastoma has an equal male-to-female ratio in our study, while both types of lymphomas, Hodgkin’s and non-Hodgkin’s, have approximately male-to-female ratio of 3.4 and 6.3, respectively. Male predominance in these lymphomas are well-documented,[2–4,6,9] although the wide male-to-female ratio may be due to the total number of cases presenting to the hospital.

Nephroblastoma has been a major childhood malignancy in our setting as per the present report.[7]

Bone tumors were low in number when compared to other soft tissue tumors. Ewing’s sarcoma has been consistently low in most African studies.[10,11]

Germ cell tumors of testicular, ovarian, and other sites are low in our study. This is similar to another report.[12] However, they play a major part in morbidity and mortality, because when present in this age group they are likely to be highly malignant.[1] Rhabdomyosarcoma is the most prevalent soft tissue sarcoma in this study. Regarding carcinomas that accounted for 2.0% of all childhood malignancies, the colorectum, conjunctiva, skin, and nasopharynx, are mainly affected. Another report shows skin as an important organ for childhood cancer.[12] Generally mesenchymal tumors are more common than epithelial tumors in childhood,[2,3,7,9,12] as in the present report.

In conclusion, lymphomas are the most common malignancies of childhood seen in this region and the majority are of the Burkitt type. This is in contrast to the predominant leukemic and central nervous system trend seen in developed nations.
